# EGR3 Promotes Glioblastoma Cell Growth with Upregulation of MYC and CDK1

**DOI:** 10.3390/ijms26135931

**Published:** 2025-06-20

**Authors:** Chia-Wei Chang, Yi-Chin Chou, Yin-Cheng Huang, Yi-Chuan Cheng

**Affiliations:** 1 Graduate Institute of Biomedical Sciences, College of Medicine, Chang Gung University, Taoyuan 333323, Taiwan; 2 Department of Neurosurgery, Chang Gung Memorial Hospital at Linkou Medical Center, Taoyuan 333423, Taiwan; 3 College of Medicine, Chang Gung University, Taoyuan 333323, Taiwan; 4 Neuroscience Research Center, Chang Gung Memorial Hospital, Linkou, Taoyuan 333423, Taiwan

**Keywords:** glioblastoma, transcriptomic analysis, EGR3, transcription factors

## Abstract

Glioblastoma (GBM) is the most aggressive and lethal primary brain tumor, characterized by rapid growth and resistance to therapy. Despite extensive research, the molecular mechanisms driving GBM progression remain incompletely understood. In this study, we employed integrative transcriptomic analysis to identify transcription factors associated with GBM, revealing EGR3 as a key candidate. Functional assays demonstrated that EGR3 promotes GBM cell viability, with EGR3 overexpression significantly enhancing cell growth, while EGR3 disruption impaired viability. To elucidate the downstream targets of EGR3, we further performed transcriptomic analysis and identified *MYC* and *CDK1* as significantly upregulated in response to EGR3 overexpression. These results suggest that EGR3 is associated with enhanced GBM cell growth, potentially through the regulation of *MYC* and *CDK1*. Our findings provide a clear model linking EGR3 to GBM proliferation and highlight MYC and CDK1 as potential therapeutic targets. This study advances the understanding of transcription factor-associated oncogenesis in GBM and suggests that targeting EGR3 may offer a novel therapeutic strategy.

## 1. Introduction

Glioblastoma (GBM) is the most common and aggressive malignant brain tumor, originating from neural progenitors or glial precursors within the neuroepithelial tissue of the brain. Classified as a grade IV tumor according to the World Health Organization (WHO) brain tumor classification, GBM exhibits significant cellular heterogeneity, invasiveness, and resistance to conventional therapies. Apart from radiation exposure and certain genetic predispositions, the precise etiology of GBM remains poorly understood, complicating both early diagnosis and effective treatment strategies. Current standard-of-care therapy combines radiotherapy with temozolomide chemotherapy, but despite this multimodal approach, patient prognosis remains dismal, with median survival rarely exceeding 15 months post-diagnosis [1].

A major challenge in treating GBM arises from the extensive molecular heterogeneity and genetic instability observed within and between individual tumors. Comprehensive genome-wide studies have consistently identified multiple genetic alterations, including amplifications, deletions, or mutations in key oncogenes and tumor suppressors, such as *EGFR*, *NF1*, *PDGFRA*, *IDH1*, *BRAF*, *TP53*, and *PTEN*. Furthermore, dysregulated signaling pathways, notably NF-κB and PI3K–AKT–mTOR, are frequently implicated in GBM pathogenesis [1,2]. Despite extensive research, targeted therapies aimed at these molecular abnormalities have generally failed in clinical trials, highlighting the complexity and redundancy of GBM signaling networks and the urgent need for novel therapeutic targets and biomarkers [3]. Given these challenges, a deeper understanding of GBM’s molecular regulators is critical. Identifying key molecular drivers of tumorigenesis and progression can provide valuable insights into novel treatment strategies. Molecular profiling has the potential to refine diagnostic classifications, predict therapeutic responses, and improve patient outcomes through precision medicine approaches. Therefore, elucidating the complex molecular landscape underlying GBM remains a priority in neuro-oncology research.

GBM’s molecular heterogeneity poses a major barrier to the discovery of consistent and generalizable therapeutic targets. Individual transcriptomic studies frequently suffer from methodological variability, small sample sizes, and dataset-specific biases, often resulting in contradictory findings. Meta-analysis offers a solution to these challenges by integrating multiple independent transcriptomic datasets to derive consensus molecular signatures. This approach increases statistical power, enhances reproducibility, and minimizes artifacts, thereby enabling the identification of robust disease-associated genes across diverse patient populations. Focusing on transcription factors (TFs) within this framework is particularly advantageous. As central regulators of gene expression programs, TFs orchestrate numerous cellular processes, including those that drive tumor proliferation, invasion, and resistance to therapy. Perturbing TFs can therefore lead to widespread shifts in downstream signaling networks. Identifying TFs that are consistently dysregulated in GBM has the potential to uncover master regulators of glioma biology and reveal novel therapeutic opportunities. Applying meta-analysis to prioritize transcriptional regulators addresses both the need for statistical rigor and biological relevance, making it a strategic approach for studying complex tumors, such as GBM.

The early growth response (EGR) family consists of four zinc-finger transcription factors—*EGR1*, *EGR2*, *EGR3*, and *EGR4*—that regulate gene expression in response to a variety of external stimuli. These TFs play key roles in controlling cell proliferation, differentiation, apoptosis, and neural development. Among them, *EGR3* has been less extensively studied in cancer biology despite its established function in neurodevelopment and psychiatric disorders, such as schizophrenia and bipolar disorder [4]. Mechanistically, *EGR3* exerts its transcriptional effects through GC-rich response elements, and its activity is tightly regulated by negative feedback from NAB1 and NAB2 corepressors [5,6]. Although *EGR3* has been implicated in breast and gastric cancers [2], its role in GBM remains unresolved. Multiple prior studies have yielded conflicting findings. Tang et al. reported that *EGR3* was upregulated in GBM tissues and cell lines and that its knockdown suppressed proliferation, migration, and invasion while promoting apoptosis—suggesting a potential oncogenic function [7]. Conversely, Shen et al. observed that *EGR3* was downregulated in glioma tissues and that its overexpression impaired proliferation and colony formation, indicating a possible tumor-suppressive role [8]. More recently, Knudsen et al. performed large-scale immunohistochemical and spatial profiling analyses and revealed that *EGR3* expression was enriched at the tumor periphery relative to the core, a region often associated with glioma cell infiltration [2]. High *EGR3* expression was also associated with poor survival in MGMT-methylated patients, implicating *EGR3* in tumor progression and potentially in cell migration [2]. Separately, Qin et al. identified *EGR3* among six differentially expressed TFs enriched in GBM samples compared to adjacent tissues. Although *EGR3* was reported as downregulated in tumor tissues, high *EGR3* expression correlated with shorter recurrence-free survival, again suggesting a functional role in disease progression [9]. These discrepancies likely reflect the context-dependent nature of transcription factor activity. Differences in tumor subtype, cellular origin, epigenetic regulation, or the local tumor microenvironment may all influence *EGR3* function. Transcription factors often operate in complex, tissue-specific networks, and their effects can vary depending on co-regulators, expression thresholds, and spatial distribution. In light of these conflicting data and the central regulatory role of TFs in tumor biology, it is essential to reassess *EGR3* in GBM through integrated transcriptomic analysis and experimental validation. A clearer understanding of *EGR3* may reconcile existing inconsistencies and clarify whether it contributes to glioma proliferation, migration, or therapeutic resistance.

Despite accumulating evidence implicating *EGR3* in glioblastoma, its functional role remains unresolved, with previous studies reporting contradictory effects on tumor cell proliferation and progression. To address this uncertainty, we performed a meta-analysis of three independent transcriptomic datasets to identify transcription factors consistently dysregulated in GBM. Among the 36 candidates identified, *EGR3* was selected for further investigation based on its differential expression and prior implications in glioma biology. We assessed the functional impact of *EGR3* using gain- and loss-of-function approaches in GBM cell lines and performed integrative transcriptomic analysis to explore its downstream regulatory network. These findings provide insights into the transcriptional programs associated with *EGR3* and its potential contribution to GBM pathophysiology.

## 2. Results

### 2.1. Identification of GBM-Associated Transcription Factors Through Integrated Transcriptomic Analysis

**Figure 1:**
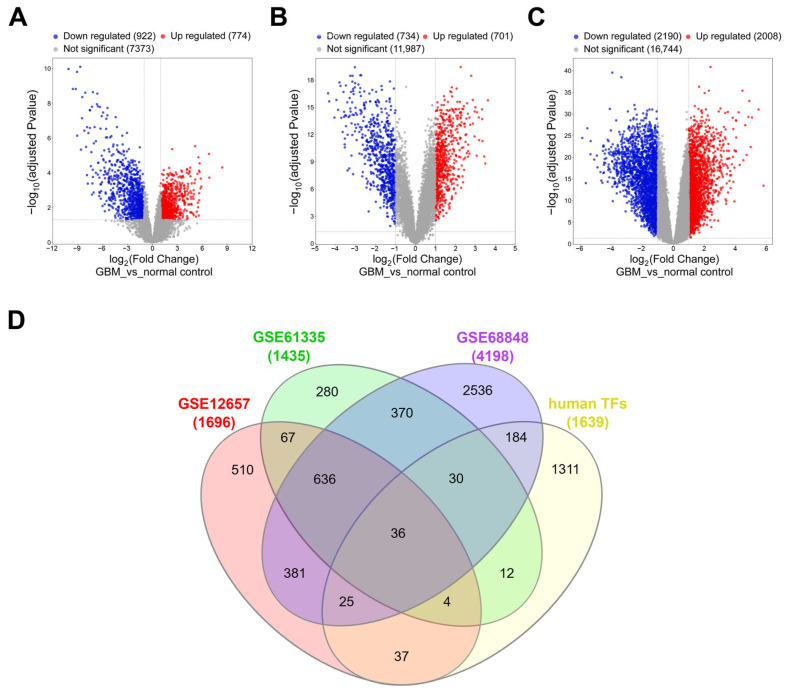
Identification of GBM-associated transcription factors through differential expression analysis. (**A**–**C**) Volcano plots displaying DEGs identified from three independent datasets: (**A**) GSE12657, (**B**) GSE61335, and (**C**) GSE68848. DEGs were defined by adjusted *p*-value < 0.05 and |log_2_ fold change| ≥ 1. Red and blue dots represent significantly upregulated and downregulated genes, respectively, while gray dots denote non-significant genes. Vertical and horizontal dashed lines indicate fold change and adjusted *p*-value thresholds. (**D**) Venn diagram showing the overlap of transcription factors (TFs) identified from the DEGs of all three datasets. A total of 36 TFs exhibited consistent differential expression across three datasets. These TFs were prioritized for further functional and survival analyses to identify GBM-associated biomarkers.

To systematically identify transcription factors involved in GBM pathogenesis, we performed an integrated transcriptomic analysis using three independent datasets from the Gene Expression Omnibus (GEO): GSE12657 (7 GBM vs. 5 control), GSE61335 (48 GBM vs. 14 normal), and GSE68848 (288 GBM vs. 28 normal). Each dataset was processed independently to preserve dataset-specific transcriptional profiles and minimize batch effects, ensuring robust and reproducible results. Differentially expressed genes (DEGs) were identified using consistent criteria across all datasets (adjusted *p*-value < 0.05 and |log_2_ fold change| ≥ 1). Specifically, GEO2R was used for DEG analysis of GSE12657 and GSE68848, while the “affy” package in R was applied for GSE61335, providing precise control over normalization and background correction for Affymetrix microarray data. This analysis yielded 1696 DEGs in GSE12657, 1435 DEGs in GSE61335, and 4198 DEGs in GSE68848. The distribution of DEGs across these datasets is visualized in volcano plots (Figure 1A–C). To focus on transcriptional regulators, the identified DEGs from each dataset were cross-referenced with a curated list of 1639 human transcription factors obtained from a publicly available database. This intersection analysis identified 36 transcription factors that consistently exhibited differential expression across all three datasets (Figure 1D). These TFs included well-known regulators of cell proliferation and differentiation, such as *MYC*, *HIF1A*, *EGR3*, and *FOXM1* (Table 1). These 36 TFs were prioritized for further analysis as candidate GBM-associated regulators.

**Figure 2:**
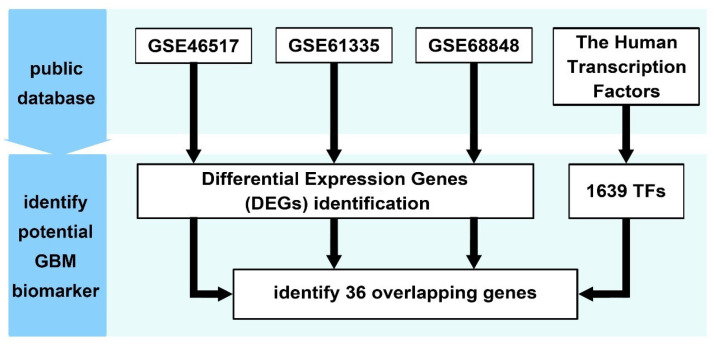
Schematic of multi-dataset transcriptomic analysis for discovering GBM-associated transcription factors.

The complete workflow of this multi-dataset transcriptomic analysis is illustrated in Figure 2. This approach, which involved independent analysis of each dataset followed by cross-referencing transcription factors, minimized dataset-specific biases while preserving the unique transcriptional characteristics of each cohort.

The schematic illustrates the integrative analysis of transcriptomic data from three GEO datasets (GSE12657, GSE61335, and GSE68848). Differentially expressed genes (DEGs) were identified independently for each dataset. GSE12657 and GSE68848 were analyzed using the GEO2R online tool, while GSE61335 was processed using the “affy” package in R, which provides precise control over background correction, normalization, and probe summarization. DEGs were cross-referenced with a curated list of 1639 human transcription factors, yielding 36 TFs consistently differentially expressed across all three datasets.

### 2.2. EGR3 Knockout Decreases GBM Cell Growth

**Figure 3:**
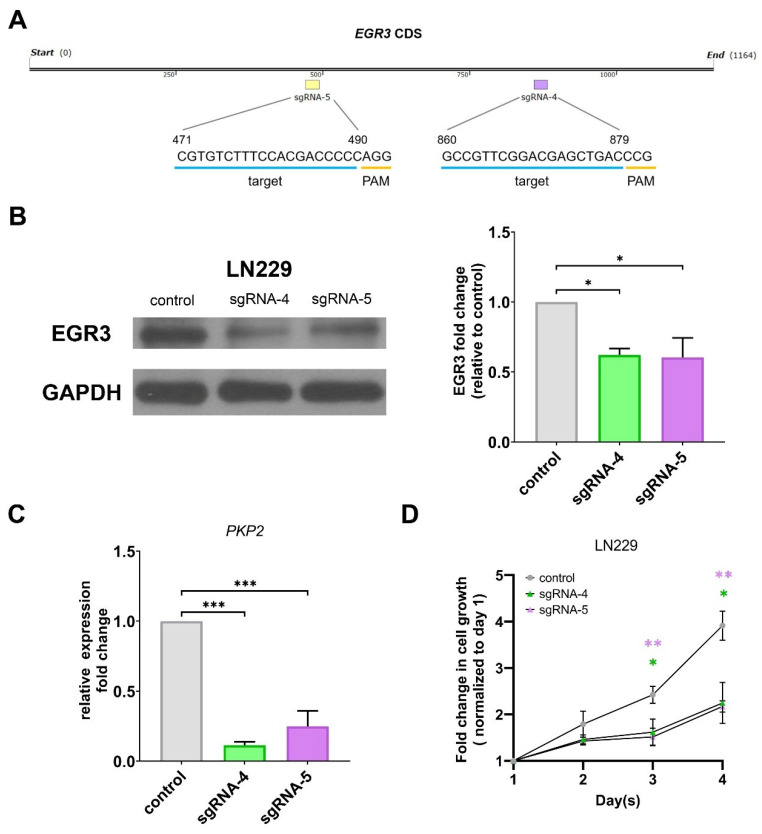
Disruption of EGR3 reduces GBM cell viability. (**A**) Schematic representation of the target sites for sgRNA-4 (nucleotides 860–879) and sgRNA-5 (nucleotides 471–490) within the *EGR3* coding region. CRISPR/Cas9-mediated gene editing was used to disrupt *EGR3* expression in GBM cell line LN229. (**B**) Western blot analysis showing partial reduction in *EGR3* protein levels in LN229 cells transfected with sgRNA-4 or sgRNA-5. GAPDH was used as a loading control. Quantification of *EGR3* protein levels was performed by densitometry and normalized to GAPDH. Data are presented as mean ± SEM. Statistical significance was assessed using one-way ANOVA followed by Dunnett’s multiple comparisons test. (**C**) Quantitative RT-PCR analysis of *PKP2* mRNA levels in LN229 cells following *EGR3* disruption. *PKP2* expression was significantly reduced compared to scramble controls, validating the effectiveness of *EGR3* disruption. Data are presented as mean ± SEM. Statistical significance was determined by one-way ANOVA followed by Dunnett’s multiple comparisons test. (**D**) MTT assay assessing cell viability in LN229 cells following *EGR3* disruption. Cells transfected with sgRNA-4 or sgRNA-5 exhibited significantly reduced MTT absorbance values compared to scramble controls, indicating decreased cell viability. Data are presented as mean ± SEM. Statistical significance was determined by two-way ANOVA followed by Bonferroni’s post hoc test. *, *p* < 0.05; **, *p* < 0.01; ***, *p* < 0.001.

To investigate the role of EGR3 in GBM cell viability, we employed CRISPR/Cas9-mediated gene editing to disrupt EGR3 expression in human GBM cell line LN229. Two independent single-guide RNAs (sgRNA-4 and sgRNA-5) targeting the coding region of EGR3 were introduced, with a scrambled sgRNA serving as a negative control (Figure 3A). Following transfection and puromycin selection, Western blot analysis confirmed a substantial reduction in EGR3 protein expression in cells transfected with either sgRNA-4 or sgRNA-5, while the scrambled control exhibited no reduction in EGR3 expression (Figure 3B). Although EGR3 protein levels were not completely abolished, the observed reduction was sufficient to assess its functional relevance in GBM cells. To confirm the effectiveness of EGR3 disruption, we assessed the expression of plakophilin 2 (PKP2), a gene previously reported as a transcriptional target of EGR3 in GBM [7]. Quantitative RT-PCR (qRT-PCR) revealed a significant reduction in PKP2 mRNA levels in EGR3-disrupted cells compared to controls, supporting that the partial reduction in EGR3 was functionally sufficient to alter downstream gene expression (Figure 3C).

We next evaluated whether *EGR3* disruption affected cell viability using MTT assays. LN229 cells transfected with sgRNA-4 or sgRNA-5 exhibited significantly lower MTT absorbance values compared to scrambled controls, indicating reduced cell viability following *EGR3* disruption (Figure 3D). These findings demonstrate that even partial suppression of *EGR3* is sufficient to compromise GBM cell viability.

While our study demonstrates that EGR3 depletion impairs GBM cell viability, we acknowledge a limitation in the extent of EGR3 suppression achieved by the CRISPR/Cas9 system. Western blot analysis revealed only a partial reduction in EGR3 protein levels, which may underestimate the full biological consequences of complete EGR3 loss. This partial depletion could reflect technical limitations of CRISPR editing efficiency or clonal heterogeneity in transfected populations. Despite this, we observed consistent reductions in cell viability and *PKP2* expression, supporting the functional relevance of EGR3 modulation. Future studies employing complementary gene-silencing approaches (e.g., inducible CRISPR systems or RNA interference) or clonal selection may help delineate the full spectrum of EGR3-mediated effects in GBM cells.

### 2.3. EGR3 Overexpression Promotes GBM Cell Growth

**Figure 4:**
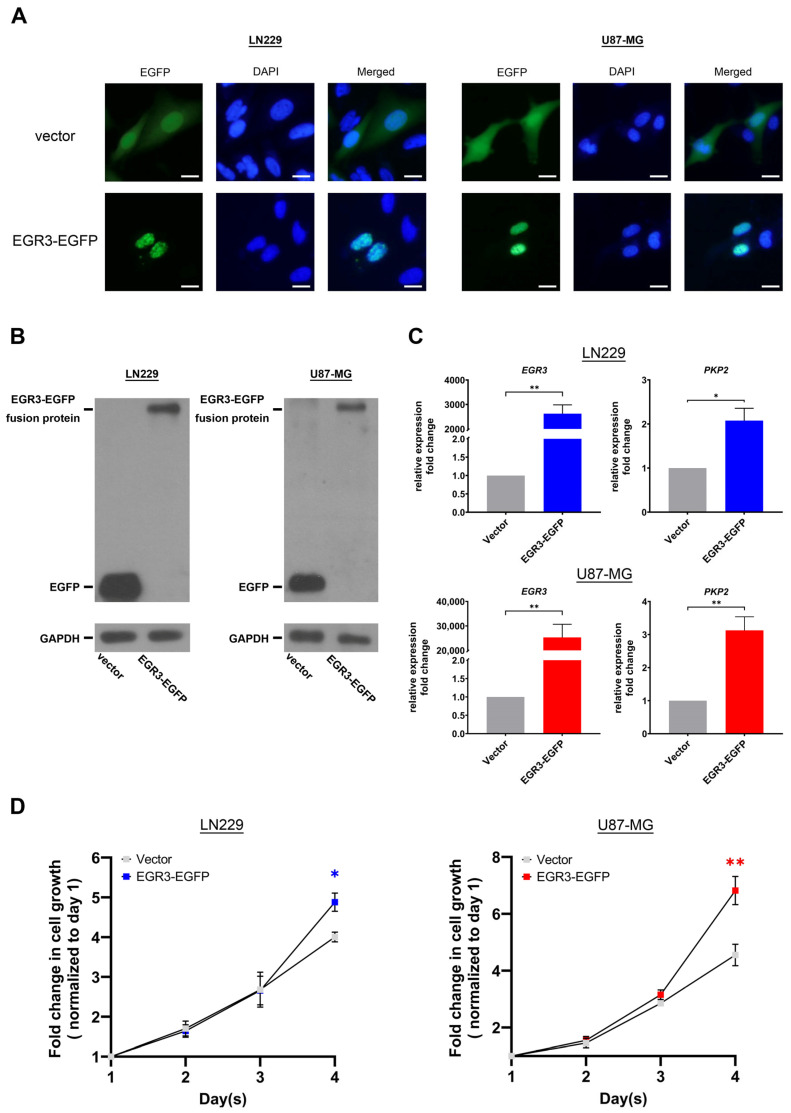
EGR3 overexpression enhances GBM cell growth. (**A**) Fluorescence microscopy images showing subcellular localization of *EGR3*-GFP and GFP control proteins in LN229 and U87-MG cells. DAPI was used to stain cell nuclei. *EGR3*-GFP fluorescence was predominantly localized within the nucleus, consistent with its role as a transcription factor, while GFP alone was diffusely distributed throughout the cytoplasm and nucleus. In panels (**A**), each scale bar represents 10 μm. (**B**) Western blot analysis confirming the expression of *EGR3*-GFP (approximately 75 kDa) and GFP (approximately 25 kDa) in LN229 and U87-MG cells using an anti-GFP antibody. The observed size of *EGR3*-GFP aligns with the expected size of the fusion protein, supporting the integrity of the construct. GAPDH was used as a loading control. (**C**) Quantitative RT-PCR (qRT-PCR) analysis demonstrating significantly increased *EGR3* mRNA levels in *EGR3*-GFP-transfected cells compared to GFP controls, confirming successful overexpression. PKP2 expression was also significantly upregulated, indicating that the overexpressed *EGR3* retained transcriptional activity. Data are presented as mean ± SEM. Statistical significance was assessed using a two-tailed Student’s *t*-test. (**D**) MTT assay assessing cell viability in LN229 and U87-MG cells following *EGR3* overexpression. Cells expressing *EGR3*-GFP exhibited significantly higher MTT absorbance values compared to GFP controls, indicating enhanced cell viability. Data are presented as mean ± SEM. Statistical significance was determined using two-way ANOVA followed by Bonferroni’s post hoc test. *, *p* < 0.05; **, *p* < 0.01.

To further investigate the role of *EGR3* in GBM, we examined the effects of *EGR3* overexpression. A plasmid encoding an *EGR3*-GFP fusion protein was constructed, enabling both overexpression and fluorescent tracking. LN229 and U87-MG cells were transfected with either the *EGR3*-GFP construct or a control vector expressing GFP alone. Fluorescence microscopy demonstrated that *EGR3*-GFP localized predominantly within the nucleus, consistent with the expected distribution of a transcription factor (Figure 4A). Western blot analysis using an anti-GFP antibody confirmed the expression of the *EGR3*-GFP fusion protein at approximately 75 kDa, consistent with the predicted size of *EGR3* (approximately 50 kDa) fused to GFP (25 kDa). This result verified that the overexpressed *EGR3* was produced as an intact fusion protein of the expected size, supporting the validity of the construct (Figure 4B). GAPDH was used as a loading control to normalize EGR3 protein levels across samples. While widely employed for this purpose, GAPDH expression may be affected under certain cancer-related metabolic conditions, and its stability should be interpreted with caution [10]. Nonetheless, in our experimental setting, GAPDH signals were consistent across groups. Quantitative RT-PCR (qRT-PCR) analysis confirmed significantly elevated *EGR3* mRNA levels in *EGR3*-GFP-transfected cells compared to GFP controls, verifying successful overexpression (Figure 4C). We next measured *PKP2* expression as a readout of *EGR3* transcriptional activity. *PKP2* expression was significantly upregulated in *EGR3*-overexpressing cells compared to GFP controls, demonstrating that the overexpressed *EGR3* protein retained its transcriptional function (Figure 4C).

We then evaluated the impact of *EGR3* overexpression on cell viability using MTT assays. LN229 and U87-MG cells transfected with *EGR3*-GFP exhibited significantly higher MTT absorbance values compared to GFP controls, indicating enhanced cell viability (Figure 4D). These findings demonstrate that *EGR3* overexpression promotes GBM cell growth, complementing our results from *EGR3* disruption experiments. The contrasting effects of *EGR3* disruption and overexpression highlight its role as a positive regulator of GBM cell viability.

### 2.4. Identification of EGR3 Downstream Targets in GBM

**Figure 5:**
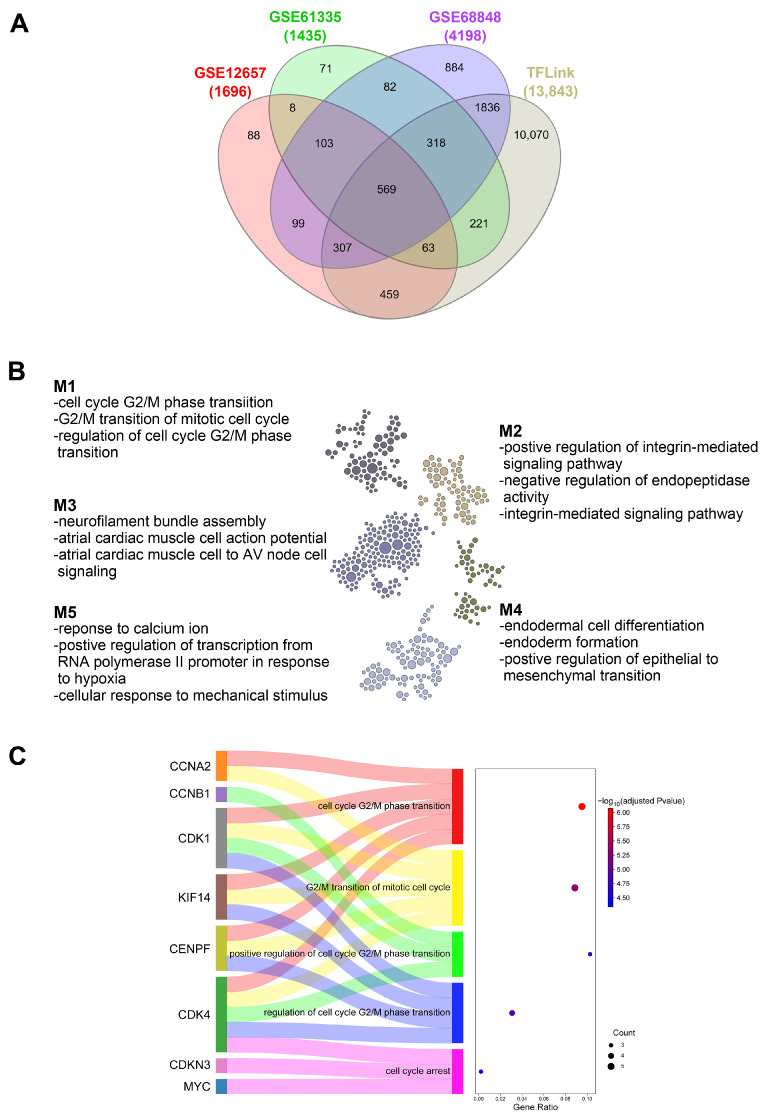
Identification of potential EGR3 downstream targets through integrative transcriptomic analysis. (**A**) Venn diagram showing the overlap between differentially expressed genes (DEGs) identified from three independent datasets (GSE12657, GSE61335, and GSE68848) and putative EGR3 target genes curated from the TFLink database. A total of 569 overlapping genes were identified as candidate downstream targets of EGR3. (**B**) Functional module classification of the 569 candidate genes using the HumanBase platform. Each dot represents an individual gene, with different colors indicating membership within distinct co-expression modules (M1–M5). Module M1, enriched for cell cycle-related processes, was prioritized for further analysis. (**C**) Sankey and dot plot illustrating the top five significantly enriched Gene Ontology biological process (GO-BP) terms identified within module M1. Eleven candidate genes involved in cell cycle-related processes were mapped. In the Sankey plot, ribbons link genes (**left**) to their associated GO terms (**right**), with colors indicating distinct biological process categories. In the dot plot, dot size represents the number of genes per term, and color intensity reflects statistical significance (−log_10_ adjusted *p*-value).

To confirm the oncogenic potential of EGR3, we next sought to elucidate its downstream targets. DEGs identified from three independent transcriptomic datasets (GSE12657, GSE61335, and GSE68848) were cross-referenced with putative transcriptional targets of EGR3 curated from the TFLink database [11], which integrates transcription factor–target gene interactions from experimentally validated sources. This intersection yielded 569 overlapping genes, representing potential mediators of EGR3-driven transcriptional programs in GBM (Figure 5A). To further characterize these potential targets, we performed Functional Module Discovery analysis using the HumanBase platform, focusing on brain-specific co-expression patterns. This analysis stratified the 569 genes into five distinct co-expression modules (M1–M5), each enriched for unique biological processes. Notably, genes in module M1 exhibited strong enrichment for biological process terms related to cell cycle regulation, particularly the G2/M phase transition—a critical checkpoint for cell proliferation. Given that EGR3 disruption reduced cell viability (Figure 3) and EGR3 overexpression enhanced cell growth (Figure 4), the strong association of M1 genes with cell cycle regulation directly aligns with our functional data, suggesting that EGR3 may promote GBM cell viability through cell cycle regulation (Figure 5B). We prioritized module M1 for further analysis, as it is directly linked to cell proliferation—a key feature of oncogenic transformation. Within module M1, we focused on genes contributing to the top enriched BP terms, including the cell cycle G2/M phase transition, the G2/M transition of mitotic cell cycles, regulation of the cell cycle G2/M phase transition, and positive regulation of the cell cycle G2/M phase transition. This refinement resulted in a subset of 8 genes (*CCNA2*, *CCNB1*, *CDK1*, *CDK4*, *CDKN3*, *CENPF*, *KIF14*, and *MYC*) that are directly involved in cell cycle progression (Figure 5C). These findings indicate that EGR3 may regulate a transcriptional network associated with cell cycle control, particularly the G2/M phase transition.

### 2.5. MYC and CDK1 Were Identified as the Primary Downstream Targets

**Figure 6:**
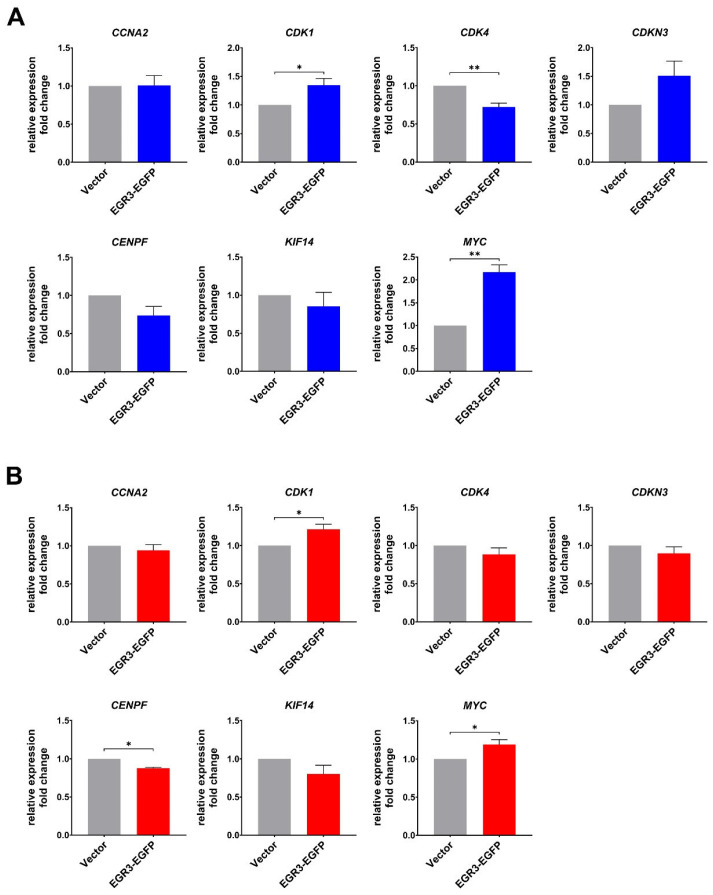
EGR3-dependent upregulation of *MYC* and *CDK1* validated by qRT-PCR. qRT-PCR analysis of the 7 candidate genes derived from module M1 in LN229 (**A**) and U87-MG (**B**) cells overexpressing EGR3. *MYC* and *CDK1* exhibited significant upregulation compared to control cells, while the remaining genes showed no significant alteration. Data represent mean ± SEM from three independent experiments. Statistical significance was determined using a two-tailed Student’s *t*-test. * *p* < 0.05; ** *p* < 0.01.

To validate the potential downstream targets identified through integrative transcriptomic analysis, we performed quantitative RT-PCR (qRT-PCR) on the 8 candidate genes derived from module M1 in LN229 and U87-MG cells overexpressing EGR3. Among these genes, only *MYC* (previously known as *c-Myc* or *MycC*) and *CDK1* showed consistent and significant upregulation upon EGR3 overexpression in both cell lines, aligning with the transcriptomic findings (Figure 6). The expression of the remaining genes (*CCNA2*, *CDK4*, *CDKN3*, *CENPF*, and *KIF14*) either showed no significant changes or displayed inconsistent patterns between U87 and LN229 cells. *CCNB1* was not detected in either cell line, likely due to expression levels below the detection threshold. These results suggest that *MYC* and *CDK1* are the primary downstream effectors regulated by EGR3 in GBM cells. The upregulation of *MYC* and *CDK1* supports their roles as mediators of EGR3-driven cell proliferation, aligning with our previous observations that EGR3 promotes GBM cell viability. These findings establish a mechanistic link between EGR3 activity and cell cycle progression, highlighting *MYC* and *CDK1* as critical effectors in this process.

## 3. Discussion

This study investigates the role of EGR3 in GBM, an aggressive and treatment-resistant brain tumor. Through integrative transcriptomic analysis of public datasets, EGR3 was identified as a transcription factor consistently associated with GBM. Functional assays demonstrated that EGR3 promotes GBM cell viability and growth. By combining transcriptomic data with experimental validation, this study provides a systematic approach to characterizing transcriptional regulators relevant to GBM pathobiology.

Our findings indicate that EGR3 promotes glioblastoma GBM cell growth, accompanied by increased expression of two oncogenic regulators, MYC and CDK1. Both genes are frequently overexpressed in GBM and are known to contribute to tumor cell proliferation. MYC functions as a key transcriptional regulator of cell growth and metabolic reprogramming and has been consistently associated with high-grade gliomas, including GBM [12]. CDK1, a central mediator of the G2/M phase transition, plays a direct role in mitotic entry and has been linked to poor prognosis in GBM [13]. The observed upregulation of *MYC* and *CDK1* following EGR3 overexpression suggests that EGR3 may influence cell proliferation by modulating transcriptional programs governing cell cycle progression and metabolism. Together, these results support the existence of an EGR3-associated regulatory program involving MYC and CDK1, while also suggesting that additional downstream targets may contribute to the broader oncogenic landscape in GBM. Although MYC and CDK1 were prioritized based on their consistent upregulation and well-established roles in glioma biology, it is likely that EGR3 governs a wider transcriptional network influencing multiple aspects of tumor progression. Additional candidate targets identified from transcriptomic analysis may play important roles in processes such as invasion, resistance to apoptosis, or therapy response. Future investigations using genome-wide approaches—such as chromatin immunoprecipitation sequencing (ChIP-seq) or transcriptomic profiling following EGR3 perturbation—will be critical to fully define the EGR3 regulatory network and its multifaceted role in GBM progression.

Although this study supports the role of EGR3 as an oncogenic driver in GBM by promoting cell growth and upregulating *MYC* and *CDK1*, its function in cancer appears to be complex and context-dependent. Previous studies have reported contradictory findings regarding EGR3′s function in gliomas. For instance, Tang et al. [7] demonstrated that EGR3 promotes GBM cell proliferation, migration, and invasion, consistent with our findings. In contrast, Shen et al. [8] reported that EGR3 acts as a tumor suppressor, inhibiting glioma cell proliferation. These conflicting results may be attributed to several factors. First, the cellular context, including differences in cell type, genetic background, and tumor microenvironment, may influence EGR3′s functional role. In some contexts, EGR3 may activate growth-promoting genes, such as MYC and CDK1, as demonstrated in this study, while in others, it may activate or repress genes involved in growth suppression or apoptosis. Second, EGR3 may participate in complex regulatory networks with context-dependent co-factors. As a member of the early growth response gene family, a group of transcription factors known for their context-dependent functions, EGR3 can either activate or repress a diverse array of target genes. The availability of co-factors, chromatin states, and interactions with other signaling pathways can dramatically alter its regulatory role. Finally, the apparent paradox may also reflect differences in experimental design, including the use of distinct cell lines, gene editing methods, and culture conditions. Our study specifically focused on human GBM cell lines, where EGR3 consistently promoted cell viability and growth. These findings may not be generalizable to other tumor types or non-cancerous cells, where EGR3 may function differently. This context-dependent regulatory role of EGR3 highlights the importance of carefully considering experimental models and conditions when interpreting its function. Our results contribute to a more nuanced understanding of EGR3, demonstrating that it can function as an oncogenic driver in the specific context of GBM, while also recognizing that its role may vary in other settings.

A deeper understanding of the conflicting roles of EGR3 in gliomas requires careful consideration of the molecular context in which EGR3 operates. Differences in glioma subtypes, such as classical versus mesenchymal, may result in divergent transcriptional programs and responsiveness to EGR3. Moreover, the tumor microenvironment—including the presence of immune cells, stromal components, and hypoxic conditions—can influence transcription factor function through paracrine signaling and chromatin remodeling. EGR3′s activity is also shaped by co-regulatory proteins, such as other transcription factors or chromatin modifiers, which may differ in abundance or interaction patterns across tumor models. These factors may determine whether EGR3 functions as a transcriptional activator or repressor of oncogenic or tumor-suppressive targets. Furthermore, post-translational modifications of EGR3, such as phosphorylation, could modulate its DNA-binding affinity and transcriptional output in a context-specific manner. Dissecting these layers of regulation in future studies will be essential to reconcile the contradictory findings and define the precise role of EGR3 in gliomagenesis.

Emerging evidence from other tumor types supports the highly context-dependent function of EGR3. In nasopharyngeal carcinoma, EGR3 is activated under hypoxia and contributes to immunosuppression and tumor growth through regulation of IL10 and TGFB1 in regulatory B cells [14]. In contrast, in breast cancer cells treated with a curcumin analog, EGR3 upregulation was linked to inhibition of cell migration, implicating a tumor-suppressive role [15]. Similarly, in hepatocellular carcinoma [16], canine mammary cancer [17], and prostate cancer [18], EGR3 appears to inhibit growth and metastasis, often through suppression of the epithelial–mesenchymal transition or induction of apoptosis-related pathways. A distinct mechanism was also reported in hepatocellular carcinoma, where EGR3 promoted Fas ligand expression to drive apoptosis [16]. However, in tamoxifen-resistant breast cancer, EGR3 was upregulated and directly promoted expression of *MCL1*, contributing to therapy resistance [19]. These results suggest that EGR3 may shift between pro- or anti-tumorigenic functions depending on the oncogenic signaling landscape or therapeutic context. Pan-cancer transcriptomic analyses further reinforce the importance of tumor context in determining EGR3 function. A comprehensive bioinformatics study revealed that EGR3 expression is altered across multiple cancers and correlates with immune infiltration, tumor mutational burden, and clinical prognosis [20]. In nasopharyngeal [21] and liver cancer [22], oncogenic microRNAs, such as miR-483-5p and miR-210, directly target EGR3 to promote tumor progression and metastasis. In leukemia, EGR3 expression was shown to be elevated at relapse and associated with immune and lineage differentiation pathways [23,24]. In prostate cancer, EGR3 suppresses metastasis by inducing the expression of tumor suppressor genes, such as ZFP36 and SOCS3 [18], while additional evidence suggests that EGR3 loss correlates with relapse and reduced survival [25]. A study in gastric cancer also reported that decreased EGR3 expression is associated with poor prognosis, further confirming its suppressive potential in some tumor types [26]. Collectively, these findings underscore that EGR3 function is shaped by a convergence of tumor-intrinsic and -extrinsic factors—including genetic alterations, transcriptional networks, hormonal cues, and microenvironmental signals. This mechanistic diversity necessitates careful delineation of EGR3′s role in each tumor setting. Future research employing integrative multi-omics approaches across defined cancer subtypes will be critical to identify shared and unique EGR3-driven pathways, ultimately informing context-appropriate therapeutic strategies.

While this study provides valuable mechanistic insights into the role of EGR3 in GBM, a key limitation is the reliance on in vitro models. The functional assays were conducted in established human GBM cell lines, which, although widely used and experimentally tractable, do not fully recapitulate the complex tumor microenvironment, cellular heterogeneity, and immune interactions observed in patient-derived glioblastomas. As such, the translatability of our findings to clinical settings remains to be validated. In future work, in vivo models—such as orthotopic xenografts or genetically engineered mouse models—will be essential to evaluate the impact of EGR3 modulation on tumor growth, invasion, and therapeutic response within the native brain microenvironment. These models will also provide a platform to assess the feasibility of targeting the EGR3–MYC–CDK1 regulatory axis as a potential therapeutic strategy. Furthermore, incorporating patient-derived glioma stem-like cells may help to better reflect interpatient heterogeneity and therapeutic resistance mechanisms.

## 4. Materials and Methods

### 4.1. Transcriptomic Dataset Collection and DEG Analysis

Three independent transcriptomic datasets, GSE12657, GSE61335, and GSE68848, were retrieved from the Gene Expression Omnibus (GEO) database. GSE12657 and GSE68848 were analyzed using the GEO2R web tool (National Center for Biotechnology Information, Bethesda, MD, USA), while GSE61335 underwent preprocessing and differential expression analysis using the “affy” package (version 1.82.0, Technical University of Denmark, Kongens Lyngby, Denmark) in R. For all datasets, differentially expressed genes (DEGs) were defined by an adjusted *p*-value < 0.05 and an absolute log_2_ fold change (|log_2_FC|) ≥ 1. DEGs were cross-referenced with a curated list of 1639 human transcription factors to identify consistently dysregulated TFs across all three datasets.

### 4.2. Survival Analysis

Survival associations for selected EGR3 target genes were evaluated using Kaplan–Meier data from the Human Protein Atlas. Gene expression levels were stratified into high and low expression groups, and survival curves were generated using the SRplot online analysis platform (Central South University, Changsha, Hunan, China). Log-rank tests were used to assess statistical significance.

### 4.3. Cell Culture and EGR3 Overexpression

LN229 and U87-MG human glioblastoma cell lines were maintained in high-glucose Dulbecco’s Modified Eagle Medium (DMEM; Gibco, Waltham, MA, USA; contributor: UNI-ONWARD Corp., New Taipei City, Taiwan) supplemented with 10% fetal bovine serum (FBS; Avantor, Radnor, Pennsylvania, USA; contributor: Blossom Biotechnologies, Inc., Taipei City, Taiwan), a 1% antibiotic-antimycotic solution (Gibco, Waltham, MA, USA; contributor: UNI-ONWARD Corp., New Taipei City, Taiwan), and 4.5 g/L of D-glucose. Cells were incubated at 37 °C in a humidified atmosphere containing 5% CO_2_. For overexpression experiments, cells were transfected with a plasmid encoding *EGR3*-GFP or a GFP control using the DreamFect Gold transfection reagent (OZ Biosciences, Marseille, France; contributor: Harmony Biosolution Inc., New Taipei City, Taiwan).

### 4.4. CRISPR/Cas9-Mediated Knockout of EGR3

Two single-guide RNAs (sgRNAs) targeting the EGR3 coding sequence were designed using the CHOPCHOP online tool [27]. sgRNA-4 (5′-GCCGTTCGGACGAGCTGACC-3′, targeting nucleotides 860–879) and sgRNA-5 (5′-CGTGTCTTTCCACGACCCCC-3′, targeting nucleotides 471–490) were individually cloned into a plasmid vector encoding both Cas9 and EGFP. LN229 cells were transfected with the sgRNA constructs using the DreamFect Gold transfection reagent, following the manufacturer’s protocol. After transfection, cells were subjected to puromycin (1 μg/mL) selection until control cells (DreamFect-only group) were completely eliminated, typically within one week. A scramble sgRNA (5′-GCTTAGTTACGCGTGGACGA-3′) was used as a negative control [28]. Knockout efficiency of EGR3 was validated by evaluating the expression of PKP2, a known downstream target of EGR3, using quantitative real-time PCR (qRT-PCR). The sequences of primers utilized in this study are provided in Table 1.

### 4.5. Western Blot Analysis

Protein lysates were prepared using a RIPA buffer supplemented with protease (Sigma-Aldrich, St. Louis, MO, USA; contributor: UNI-ONWARD Corp., New Taipei City, Taiwan) and phosphatase inhibitors (EMD Millipore Corp., Burlington, MA, USA; contributor: UNI-ONWARD Corp., New Taipei City, Taiwan). Samples were separated on SDS-PAGE gels and transferred onto PVDF membranes (EMD Millipore Corp., Burlington, MA, USA; contributor: UNI-ONWARD Corp., New Taipei City, Taiwan). Membranes were probed with primary antibodies against EGR3 (1:6000; Cloud-Clone Corp., Houston, TX, USA; contributor: ASIA-BIOSCIENCE CO., LTD., Taipei City, Taiwan), GFP (1:5000; Proteintech Group, Rosemont, IL, USA; contributor: ABreal Biotech Co., Taipei City, Taiwan), and GAPDH (1:15,000; Proteintech Group, Rosemont, IL, USA; contributor: ABreal Biotech Co., Taipei City, Taiwan), followed by HRP-conjugated secondary antibodies. Signal detection was performed using enhanced chemiluminescence (ECL; Cytiva, Marlborough, Massachusetts, USA; contributor: UNI-ONWARD Corp., New Taipei City, Taiwan).

### 4.6. Quantitative Real-Time PCR (qRT-PCR)

Total RNA was extracted using the TRIzol reagent (Invitrogen, Carlsbad, CA, USA; contributor: Life Technologies Co., Ltd., Taipei City, Taiwan) and reverse-transcribed into cDNA using the First Strand Synthesis Kit (BIONOVAS Biotechnology Co., Ltd., Seattle, WA, USA; contributor: WonWon Biotechnology Co., Ltd., Taoyuan City, Taiwan). Quantitative real-time PCR was performed using the SYBR Green Master Mix (ABclonal, Woburn, Massachusetts, USA; contributor: BIOTOOLS CO., LTD., New Taipei City, Taiwan) on an ABI system (Applied Biosystems, Thermo Fisher Scientific, Foster City, CA, USA; contributor: LIFE TECHNOLOGIES CO., LTD., Taipei City, Taiwan). *GAPDH* served as the internal control. Gene expression levels were calculated using the 2^−ΔΔCt^ method.

### 4.7. Cell Viability Assay

Cell viability was evaluated using the MTT assay following *EGR3* overexpression or knockout. Briefly, 5 × 10^3^ cells per well were seeded into 96-well plates in 200 μL of a complete medium and incubated overnight. Subsequently, 10 μL of the MTT reagent (5 mg/mL) was added directly to each well and incubated for 3 h at 37 °C. After incubation, the supernatant was carefully removed, and 100 μL of DMSO was added to dissolve the formazan crystals. Absorbance was measured at 570 nm, and viability was compared between control and experimental groups.

### 4.8. Transcriptomic Integration and Functional Enrichment

Putative *EGR3* targets were retrieved from the TFLink database [11] and cross-referenced with DEGs identified in the meta-analysis. The overlapping gene set was analyzed using HumanBase for functional module discovery with brain-specific settings [29]. Gene ontology biological process enrichment analysis was performed to identify regulatory modules. A subset of cell cycle-related genes from the top-enriched module was selected for further analysis.

### 4.9. Statistical Analysis

Statistical analyses were performed using GraphPad Prism version 9.0 (GraphPad Software, Boston, MA, USA) and the SRplot online analysis platform [30]. qRT-PCR results were analyzed using either one-way analysis of variance (ANOVA) with Dunnett’s post hoc test or a two-tailed unpaired Student’s *t*-test, depending on the number of comparison groups. Western blot quantification data were evaluated using one-way ANOVA followed by Dunnett’s multiple comparisons test. Cell viability results from MTT assays were analyzed using two-way ANOVA with Bonferroni correction. Data are presented as means ± standard errors of the mean (SEM). Statistical significance was defined as follows: *, *p* < 0.05, **, *p* < 0.01, ***, *p* < 0.001, and ****, *p* < 0.0001.

## Figures and Tables

**Table 1:** Transcription factors consistently differentially expressed across three GBM transcriptomic datasets.

*BCL11A*	*FOXD1*	*MBNL2*	*NR3C2*	*SOX4*	*YBX3*
*CAMTA1*	*FOXM1*	*MEF2C*	*PBX3*	*STAT4*	*ZBTB18*
*CAMTA2*	*HIF1A*	*MYC*	*PLSCR1*	*TBR1*	*ZBTB20*
*CUX2*	*HIVEP2*	*MYRF*	*PRRX1*	*TCF12*	*ZIC1*
*E2F5*	*HLF*	*MYT1L*	*SATB1*	*TGIF1*	*ZNF217*
*EGR3*	*MBD2*	*NEUROD2*	*SOX10*	*YBX1*	*ZNF365*

## Data Availability

The data that support the findings of this study are available from the corresponding author upon reasonable request.

## Abbreviations

The following abbreviations are used in this manuscript:

GBM	Glioblastoma
TF	Transcription factor
DEG	Differentially expressed gene
GEO	Gene Expression Omnibus
qRT-PCR	Quantitative real-time polymerase chain reaction
GFP	Green fluorescent protein
MTT	3-(4,5-dimethylthiazol-2-yl)-2,5-diphenyltetrazolium bromide
DMEM	Dulbecco’s Modified Eagle Medium
FBS	Fetal bovine serum
SEM	Standard error of the mean
ANOVA	Analysis of variance
PVDF	Polyvinylidene difluoride
ECL	Enhanced chemiluminescence
